# Anti-Muscle Specific Kinase (Anti-MuSK) Positive Myasthenia Gravis Overlapping With Parkinson’s Disease: A Challenging Diagnosis

**DOI:** 10.7759/cureus.14839

**Published:** 2021-05-04

**Authors:** Mohammed S Albassam, Salman A Thabet, Mohammed Hmoud, Seraj Makkawi

**Affiliations:** 1 College of Medicine, King Saud Bin Abdulaziz University for Health Sciences, Jeddah, SAU; 2 Department of Medicine, Ministry of the National Guard-Health Affairs, Jeddah, SAU; 3 College of Medicine, University of Bisha, Bisha, SAU; 4 Research and Development, King Abdullah International Medical Research Center, Jeddah, SAU

**Keywords:** myasthenia gravis (mg), parkinson' s disease, adult neurology, anti-musk, rituximab, saudi arabia

## Abstract

The concomitance between Parkinson’s disease (PD) and myasthenia gravis (MG) is rare, with only a few case reports in the literature and only one of them with positive anti-muscle specific kinase (anti-MuSK) MG. The overlap between PD and MG symptoms can cause a diagnostic dilemma for the treating physician. In this report, we present a 73-year-old lady with a history of recurrent falls, dysphagia, and diplopia. She was found to have ptosis, vertical gaze restriction, neck extension, and flexion weakness, as well as features of parkinsonism, including masked face appearance, asymmetrical limbs rigidity, and bradykinesia. She was found to have a high titer antibody for MuSK MG. Her MG symptoms were treated successfully with rituximab.

## Introduction

Myasthenia gravis (MG) disease is an autoimmune neuromuscular junction disorder. Its incidence is around seven to 23 new cases each year per million persons [1-4]. MG has many subtypes depending on the antibodies produced by the B-cells directed against the acetylcholine receptor (AChR), muscle-specific kinase (MuSK), lipoprotein-related protein 4 (LRP4), and seronegative MG. The prevalence of each one of them is 80%, 4%, 2%, and 5%, respectively [5]. Whereas Parkinson's disease (PD) has an incidence of approximately eight to 18.6 per 100,000 persons in a year [6]. The concomitance between PD and MG is rare, and since 1987, 29 cases have been reported [7]. In this report, we present a case of positive anti-MuSK MG overlapping with PD.

## Case presentation

A 73-year-old lady presented to the emergency department for recurrent falls with head injuries. Also, she had gradual progressive dysphagia with frequent choking episodes and recurrent admissions for aspiration pneumonia over the last 18 months. Additionally, the patient complained of generalized weakness, fatigue, and slowness of her movement and gait. Upon further questioning, she had intermittent fatigable diplopia, especially at night; otherwise, she has no other aggravating or relieving factors. Her past medical history was significant for hypertension, diabetes, and dyslipidemia. Her medications included amlodipine, valsartan, metformin, and atorvastatin.

On examination, the patient was conscious, alert, and oriented. She had intermittent diplopia and ptosis. Also, she had vertical gaze restriction, weak jaw closure, and bilateral facial weakness, with a masked face appearance. Her speech was nasal and hypophonic. She had neck rigidity with significant neck extension and flexion weakness graded (2/5). Her motor examination showed evidence of Parkinsonism with moderate rigidity and bradykinesia affecting both upper and lower limbs, right more than left. Also, she had generalized weakness graded -4 to 4/5, affecting the upper limbs more than lower, proximally more than distally. She had normal reflexes and sensory, and cerebellar examination. Her gait showed evidence of short steps with stooped posture. Based on the patient's clinical presentation, parkinsonism overlapping with MG was suspected. 

**Figure 1:**
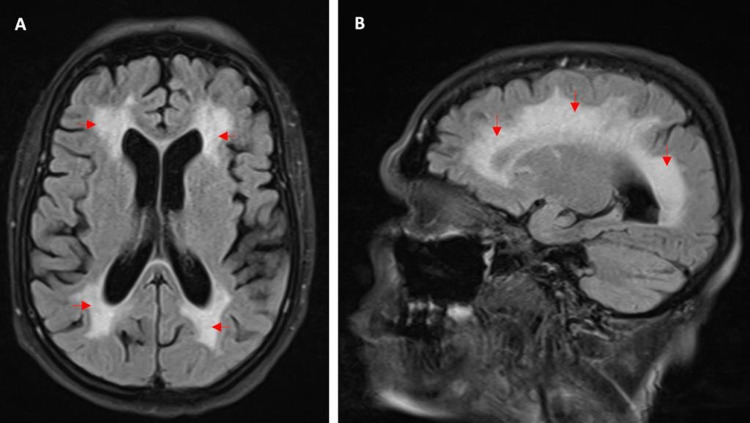
Brain MRI showing extensive chronic microangiopathic changes (A) Axial and (B) sagittal views of the brain (fluid-attenuated inversion recovery (FLAIR) sequence) showing confluent T2 high signal intensity involving the periventricular, deep and subcortical white matter, suggestive of extensive chronic microangiopathy.

Multiple investigations were performed for both diagnoses. Brain MRI showed only chronic microangiopathic ischemic changes (Figure 1). Repetitive nerve stimulation (RNS) of the ulnar nerve was normal. The patient could not tolerate RNS in other nerves or single-fiber electromyography (SFEMG). The acetylcholine receptor (Anti-AChR) antibody test was negative. However, anti-muscle specific kinase (anti-MuSK) came back highly positive at 93.4 nmol/L. Anti-ACR, anti-JO, anti-SS, anti-SM, and anti-RNP antibodies were negative. Chest CT showed no evidence of thymoma or thymic hyperplasia.

A trial of intravenous immunoglobulin (IVIG) 0.4 g/kg/day but was stopped prematurely on day two due to an allergic reaction without significant improvement. Later on, the patient developed type 2 respiratory failure requiring intubation with mechanical ventilation and admission to the intensive care unit. The patient received five sessions of plasmapheresis (PLEX) and intravenous high dose methylprednisone. A few days later, the patient improved and was extubated. She was placed on high-dose prednisone and pyridostigmine 60 mg QID. Initially, she was placed on azathioprine as maintenance but, later on, was switched to rituximab due to recurrent MG exacerbation.

On follow-up examination, she had significant improvement of her MG symptoms, including ptosis, diplopia, eye movement abnormality, and neck and limb weakness. Initially, a percutaneous endoscopic gastrostomy (PEG) tube was placed for feeding, however, a few months later, the patient tolerated oral feeding, and the tube was removed. She was started on levodopa/carbidopa for the underlining parkinsonism.

## Discussion

PD is one of the commonest neurologic diseases. It is a degenerative, progressive disease. Its prevalence is estimated to be 0.3% worldwide [8]. It is manifested by motor and nonmotor symptoms, including resting tremor, bradykinesia, postural instability, and rigidity. The most common symptom is tremor [9]. Nonmotor symptoms of PD are as follows: cognitive decline, depression, anxiety, dysautonomia, and sleep disturbances. Other gastrointestinal complaints include bloating, nausea, and abdominal discomfort [10].

Substantia nigra and locus coeruleus depigmentation with neuronal loss in the pars compacta of the substantia nigra is the pathological hallmark of PD. Meanwhile, the primary cause of PD remains unclear [11]. Regarding the treatment of PD, levodopa, which is the immediate precursor of dopamine, was the first effective medication and is still the most potent. It is mostly paired with carbidopa to lessen the peripheral side effects, particularly nausea [8]. There are other medications that can be used for PD such as dopamine agonists, which are pramipexole, ropinirole, and rotigotine [8]. Catechol-O-methyl transferase inhibitors and monoamine oxidase aldehyde dehydrogenase B inhibitors inhibit enzymes involved in the breakdown of levodopa and dopamine.

MG is a B-cell-mediated autoimmune neuromuscular junction disease characterized mainly by fatigable muscle weakness [5]. Its pathogenic autoantibodies are generated against certain components of the postsynaptic endplate [5]. Those components are AChR, MuSK, LRP4, or agrin protein in the postsynaptic membrane [5]. Moreover, antibodies against those components are considered diagnostic markers. In addition, subgrouping patients with MG using the aforementioned antibodies has an important implication on the treatment and prognosis [5].

MG patients present with muscle weakness that varies throughout the day; fatigability and exercise-induced weakness are strong clues to diagnose MG for all subtypes [5]. Besides, anti-MuSK MG shows predominant cranial and bulbar muscle involvement that is mostly accompanied by neck and respiratory involvement [5]. In addition to the antibody testing and clinical signs and symptoms, neurophysiological studies like RNS and SFEMG can help in diagnosing MG [5]. MG management is usually composed of symptomatic, immunosuppressive, supportive, and surgical treatments [5]. Acetylcholinesterase inhibitors, such as neostigmine, ambenonium chloride, and pyridostigmine, the last is the preferred symptomatic medication [5]. Immunosuppressive medications can be used for those in whom symptomatic treatment is not adequate and can be used with all MG subtypes [5]. Steroids such as prednisone or prednisolone are often used first [5]. Azathioprine is another, and the same goes for it, as it is effective in all MG subtypes, and in combination with prednisone, they are the first-line treatment [5]. Rituximab can be used as a steroid-sparing treatment for refractory patients with MG, especially patients with the anti-MuSK MG subtype [12]. Thymectomy has been reported to have an excellent response with all MG subtypes excluding anti-MuSK and LRP4.

After performing a thorough literature review based on the PubMed database using search words “Parkinson’s disease” and “Myasthenia gravis,” we found only a few case reports published worldwide. To our knowledge, there is only one published case report of anti-MuSK positive MG in a patient with parkinsonism [13]. In this report, we present another case of positive anti-MuSK MG overlapping with PD. Basically, the diagnosis of PD and MG simultaneously is often challenging due to the rareness of the concomitant of both diseases [7]. Moreover, they both share many symptoms that make the diagnosis vague and blurry, such as dysphagia, dysarthria, and eye movement, but with careful history-taking and physical examination, one can notice and differentiate the overlapping symptoms between PD and MG [7]. Muscle weakness is usually accompanied by MG and is worse by the end of the day or after repetitive movement, but patients with PD tend to have limb rigidity rather than muscle weakness [5,14-15]. Dysphagia comes in both PD and MG, especially in anti-MuSK MG since one of its first and dominant symptoms is bulbar weakness, including pharyngeal and tongue weakness [5,14-15]. In addition, dysarthria is noticed in both PD and MG, once again with anti-MuSK particularly due to the bulbar involvement [5,14-15]. Also, speech in patients with PD is monotonic and soft while MG patients typically present with nasal speech (Table 1) [5,14-15].

**Table 1:** Distinguishing clinical features of myasthenia gravis and Parkinson’s disease

Clinical features	Myasthenia Gravis	Parkinson’s Disease
Face	Facial diplegia	Masked face
Eye movement	Fatigable ophthalmoplegia	Vertical gaze palsy in progressive supranuclear palsy
Eyelid	Fatigable ptosis	Reduced blink rate
Swallowing	Fatigable dysphagia	Progressive dysphagia
Speech	Nasal speech	Soft and monotonic speech
Neck	Neck flexion/extension weakness	Neck rigidity in progressive supranuclear palsy
Limbs	Fatigable weakness	Rigidity, bradykinesia, and tremor

In the previous reports of PD overlapping with MG, the features of parkinsonism in almost all patients were treated by levodopa as our patient. All reported cases were treated by steroid alone or combined with azathioprine for MG [7]. Our patient had an excellent response to rituximab, which is typical for anti-MuSK MG.

## Conclusions

In conclusion, the overlapping symptoms between PD and MG can be challenging and should be evaluated thoroughly and carefully with a full medical history and physical examination. Although the chance of the concomitance of both diseases is very low, one should not exclude this probability since the management differs. This case report is a message for all to always think of the co-existence of PD with MG in case of overlapping symptoms for both diseases. The possibility of co-existing MG should be considered in patients with PD when distinct symptoms, such as fatigable ptosis, diplopia, or muscle weakness, develop during the disease course.
